# Update on Immune Checkpoint Inhibitors for Conjunctival Melanoma

**DOI:** 10.18502/jovr.v17i3.11579

**Published:** 2022-08-15

**Authors:** Ho-Seok Sa, Claire Daniel, Bita Esmaeli

**Affiliations:** ^1^Department of Ophthalmology, Asan Medical Center, University of Ulsan College of Medicine, Seoul, Korea; ^2^Moorfields Eye Hospital, National Health System, London, United Kingdom; ^3^Orbital Oncology and Ophthalmic Plastic Surgery, Department of Plastic Surgery, The University of Texas MD Anderson Cancer Center, Houston, Texas, USA

**Keywords:** Checkpoint Inhibitor, Conjunctival Neoplasm, Immunotherapy, Melanoma

## Abstract

The management of conjunctival melanoma is challenging due to the more frequent local recurrence and metastasis compared to other conjunctival neoplasms. Locally advanced conjunctival melanoma may require an orbital exenteration, and treatment options for metastatic conjunctival melanoma have been limited until recently. This review aims to provide comprehensive updates on immunotherapy for conjunctival melanoma, focusing on immune checkpoint inhibitors. We reviewed the available literature on the use of immunotherapy for the treatment of conjunctival melanoma. Systemic immunotherapy, particularly with checkpoint inhibitors, has recently been reported to have improved outcomes for patients with conjunctival melanoma. Immune checkpoint inhibitors that are currently approved by the US Food and Drug Administration for melanoma include anti-PD-1 (nivolumab and pembrolizumab), anti-PDL-1 (avelumab and atezolizumab), and anti-CTLA-4 inhibitors (ipilimumab). Most recent reports described using immune checkpoint inhibitors in patients with locally advanced conjunctival melanoma in an attempt to avoid orbital exenteration or in patients with metastatic conjunctival melanoma.Although the current data are limited to case reports and small case series, eye care providers should be aware of the potential role of immunotherapy for patients with locally advanced, recurrent, or metastatic conjunctival melanoma.

##  INTRODUCTION

Various neoplasms can arise in the conjunctiva from the epithelium and stroma, similar to those from other mucous membranes in the body. Initial treatment typically involves surgical excision with or without adjuvant therapies, but other nonsurgical options can now be applicable to conjunctival neoplasms. Ocular surface squamous neoplasia (OSSN) is the most common conjunctival neoplasm, with an incidence ranging from 0.03 to 1.9 per 100,000 persons/year.^[1]^ To avoid invasive surgery for the ocular surface, topical eye drops with anti-neoplastic efficacy, such as 5-fluorouracil, mitomycin-C, and interferon-α2b, have been successfully used.^[1,2]^

Conjunctival melanoma is a rare neoplasm, with an incidence of 0.054 per 100,000 persons/year.^[3]^ It can arise de novo or from a pre-existing pigmented nevus, but most commonly occurs from primary acquired melanosis (PAM).^[3,4]^ The management of conjunctival melanoma is particularly challenging due to relatively frequent local recurrence and metastasis. Despite wide excision with or without cryotherapy, which is the mainstay of treatment, conjunctival melanoma has a risk of local recurrence ranging from 18% to 83%, a risk of lymph node metastasis ranging from 15% to 41%, and a disease-specific mortality rate of about 20%.^[5,6,7,8]^ Locally advanced or recurrent conjunctival melanoma may require highly morbid surgery such as an orbital exenteration,^[3,5,9,10]^ however, orbital exenteration causes severe orbitofacial disfigurement, and it has not been shown to decrease the risks of metastasis and subsequent death.^[11]^ Metastatic conjunctival melanoma has been historically challenging to treat with poor survival outcomes despite standard systemic chemotherapy.^[7]^ Therefore, nonsurgical therapeutic options would be beneficial to avoid orbital exenteration or to treat metastatic disease. As with many other types of tumors, the immune system plays a vital role in the progression of conjunctival melanoma. More recently, there have been several positive reports of response to immunotherapy in patients with locally advanced or metastatic conjunctival melanoma. In this report, we reviewed the available literature regarding immunotherapy for conjunctival melanoma, focusing specifically on immune checkpoint inhibitors.

### Immune Checkpoint Inhibitors for Conjunctival Melanoma

Cancer progression is associated with suppression of the human immune system, especially T-cell-mediated cellular immunity. Immune checkpoints are molecules that generally help lessen the activity of cytotoxic T cells, preventing autoimmunity.^[12]^ Cancer cells can activate various immune checkpoint pathways to suppress immune responses against cancers.^[12]^ Clinically relevant checkpoints include programmed cell death-1 (PD-1) on T cells, programmed cell death ligand-1 (PDL-1) on tumor cells, and cytotoxic T lymphocyte-associated antigen-4 (CTLA-4) on T cells.^[13]^ It was recently revealed that immune checkpoints can be blocked, leading to new immunotherapeutic drugs that potentiate the patient's own cellular mediated response to attack cancer cells. Since 2011, several immune checkpoint inhibitors have been approved for patients with unresectable or metastatic melanoma by the US Food and Drug Administration (FDA), including anti-PD-1 (nivolumab and pembrolizumab), anti-PDL-1 (avelumab and atezolizumab), and anti-CTLA-4 inhibitors (ipilimumab).^[14,15,16,17]^ Notably, Dr. James P. Allison and Dr. Tasuku Honjo were awarded the 2018 Nobel Prize in Physiology or Medicine for their discovery of cancer therapy by inhibition of CTLA-4 and PD-1, respectively.

**Figure 1:**
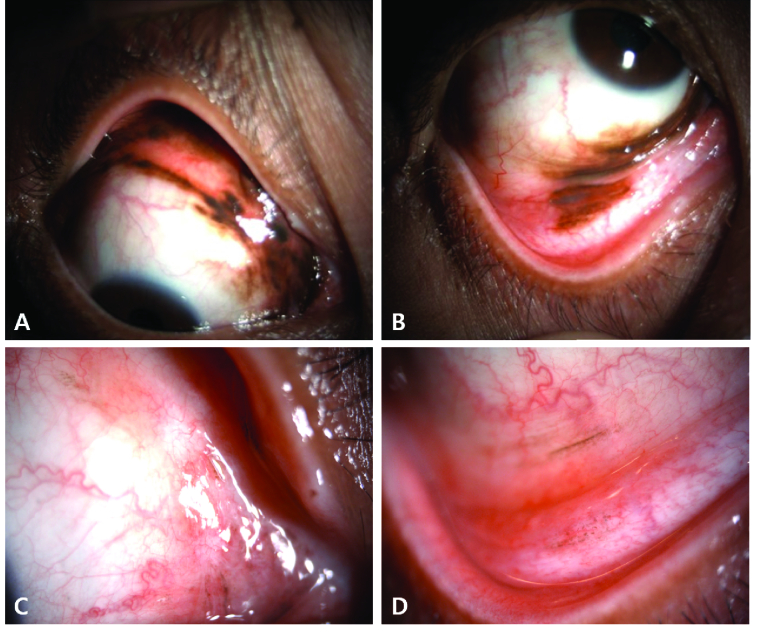
(A–D) A 55-year-old woman with locally advanced conjunctival melanoma involving multiple quadrants of the bulbar conjunctiva in addition to tarsal and palpebral conjunctiva in the upper and lower eyelids and caruncle (A, B). She was treated at M. D. Anderson Cancer Center with nivolumab (PD-1 inhibitor) for 12 cycles (over approximately a one-year period) to avoid an orbital exenteration. She had near-complete resolution of all pigmentation (C, D). The residual faint pigmentation was biopsied and found to consist of pigment-laden macrophages rather than residual melanoma. Reprinted from: Hong BY, Ford JR, Glitza IC, Cabala CAT, Tetzlaff M, Prieto VG, et al. Immune checkpoint inhibitor therapy as an eye-preserving treatment for locally advanced conjunctival melanoma. *Ophthalmic Plast Reconstr Surg* 2021;37:e9-e13.^[21]^ Copyright (2021) by Lippincott Williams & Wilkins..

Immune checkpoint inhibitors can be used for all types of melanoma including cutaneous, mucosal, and conjunctival melanomas, but they have shown variable treatment efficacy depending on different genetic features. Specifically, the tumor mutation burden is correlated with an expected response to immune checkpoint inhibitor therapy; the higher the mutation burden of cancer, the higher the likelihood of response.^[18]^ The majority of the clinical trials with immune checkpoint inhibitors have been conducted in patients with metastatic cutaneous melanoma, and data for conjunctival melanoma are limited to case reports and small case series [Table 1].^[19,20,21,22]^ However, immune checkpoint inhibitors seem to be promising for managing advanced conjunctival melanoma. This is because molecular and biologic features of conjunctival melanoma are more similar to cutaneous melanoma than mucosal melanoma, and are remarkably different from uveal melanoma.^[23]^ The use of immune checkpoint inhibitors in conjunctival melanoma generally follows similar dosing schemes as in cutaneous melanoma, as described in the US FDA reports on nivolumab (240 mg intravenously every two weeks or 480 mg every four weeks), pembrolizumab (200 mg intravenously every three weeks), and ipilimumab (3 mg/kg intravenously every three weeks).^[14,15,16,17]^ To date, most reports are on using immune checkpoint inhibitors in patients with locally advanced conjunctival melanoma in an attempt to avoid orbital exenteration [Figure 1A] or in patients with metastatic conjunctival melanoma [Figure 1B].^[19,20,21,22]^

**Figure 2:**
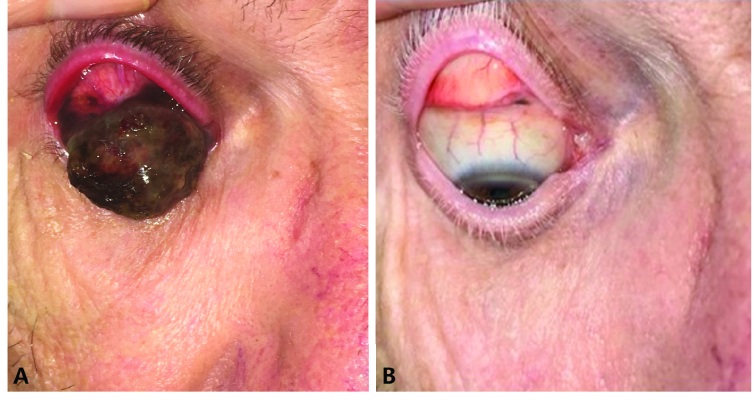
(A–B) A 66-year-old man with large invasive melanoma of bulbar and tarsal conjunctiva (A) presented to Moorfields London was found to have invasive melanoma with lung metastasis. He was treated with a combination of ipilimumab and nivolumab at Moorfields Eye Hospital for six cycles of therapy over five months. Both the eye lesion and the lung metastases responded to the treatment (B). Reprinted from: Hong BY, Ford JR, Glitza IC, Cabala CAT, Tetzlaff M, Prieto VG, et al. Immune checkpoint inhibitor therapy as an eye-preserving treatment for locally advanced conjunctival melanoma. *Ophthalmic Plast Reconstr Surg* 2021;37:e9-e13.^[21]^ Copyright (2021) by Lippincott Williams & Wilkins.

**Table 1:** List of published conjunctival melanoma cases treated with systemic immune checkpoint inhibitors


**Authors**	**Country**	**Patients (** ***n*** **)**	**Age (yrs)**	**Sex**	**Original melanoma site**	**Treatments for primary melanoma**	**Melanoma status prior to immunotherapy**	**Immune checkpoint inhibitors**	**Dosage**	**Outcomes**	**Follow-up (months)**	**Adverse effects **
Sagiv et al (2018)^[20]^	USA	5	58	F	Conjunctiva	Orbital exenteration	Recurrence at orbital rim, Systemic metastases to lungs and liver	Nivolumab	3 mg/kg every 2 wks, 6 cycles	Alive without disease	9	Mild hepatotoxicity
		28	F	Conjunctiva	Excision, cryotherapy, topical mitomycin	Metastases to breast, lungs, thigh, and clavicle	Nivolumab	3 mg/kg every 2 wks, 7 cycles	Alive without disease	36	None
		47	F	Conjunctiva	Excision, cryotherapy, brachytherapy, topical interferon	Metastasis to lungs	Nivolumab	3 mg/kg every 2 wks, 10 cycles	Alive without disease	7	Autoimmune colitis
		68	F	Conjunctiva	Orbital exenteration, irradiation	Metastasis to lungs	Pembrolizumab	2 mg/kg every 3 wks, 16 cycles	Stable disease at 6 months, then switched to ipilimumab owing to progression	2	None
				Ipilimumab	3 mg/kg every 3 wks, 2 cycles	Partial regression, Alive with disease	2	Hepatotoxicity
		74	M	Conjunctiva	Multiple resections	Metastasis to lungs	Nivolumab	3 mg/kg every 2 wks, 22 cycles	Alive without disease	1	Autoimmune colitis
Hong et al (2021)^[21]^	USA	2	53	F	Conjunctiva	Diffuse multifocal lesions	Pembrolizumab	200 mg every 3 wks, 15 cycles	Alive without disease	12	Mild cutaneous pruritus
		66	M	Conjunctiva	Locally advanced melanoma with metastases to lung and liver	Ipilimumab + Nivolumab	6 cycles	Alive without disease	9	Pituitary failure
Finger et al (2019)^[22]^	USA	5	76	M	Conjunctiva	Multiple resections, topical interferon	Diffuse multifocal recurrence	Pembrolizumab	2 mg/kg every 3 wks, 25 cycles	Alive without disease	48	Cutaneous pruritus and rash
		94	F	Conjunctiva	Diffuse multifocal lesions	Pembrolizumab 200 mg + Ipilimumab 1 mg/kg	Every 3 wks, 10 cycles	Partial regression, Dead of unrelated causes	5	None
		84	F	Conjunctiva	Multiple resections, cryotherapy, topical mitomycin, brachytherapy	Diffuse multifocal recurrence	Pembrolizumab 200 mg + Ipilimumab 1 mg/kg	Every 3wks, 15 cycles	Partial regression, Alive with disease	On immunotherapy	None
		76	F	Conjunctiva	Excision, cryotherapy, topical mitomycin	Metastases to neck, mediastinal and subcarinal lymph nodes	Ipilimumab	3 mg/kg every 3 wks, 8 cycles	No evidence of disease at 3 yrs, then metastasis to buttock	None
				Pembrolizumab (with irradiation)	200 mg every 3 wks, 8 cycles	Alive without disease	24	None
		72	F	Conjunctiva	Excision, topical chemotherapy	Metastases to lungs, liver, bone, and nodes	Ipilimumab 3 mg/kg + Nivolumab 1 mg/kg	Every 3 wks, 4 cycles	Alive without disease	36	Hepatotoxicity, colitis, pneumonitis
Kini *et al*. (2017)^26^	USA	1	60s	M	Conjunctiva	Excision, cryotherapy	Diffuse multifocal recurrence with orbital and intraocular invasion	Pembrolizumab	150 mg every 3 wks, 18 cycles	Alive without disease	On immunotherapy	None
Pinto et al (2017)^[27]^	Portugal	2	56	F	Conjunctiva	Excision	Metastasis to oropharynx and nodes	Vemurafenib	480 mg twice a day for 3 years	Alive without disease	On immunotherapy	Arthralgia,diarrhea, skin rash
		51	M	Conjunctiva	Multiple resections	Metastases to multiple nodes	Pembrolizumab	2 mg/kg every 3 wks, 13 cycles	Alive without disease	On immunotherapy	None
Chaves et al (2018)^[29]^	USA	1	72	M	Conjunctiva	Debulking, brachytherapy	Diffuse multifocal conjunctival melanoma and positive sentinel lymph node	Ipilimumab (adjuvant)	3 mg/kg every 3 wks, 4 cycles	Alive without disease	16	None
Ford et al (2017)^[19]^	USA	2	72	M	Scalp	Metastasis to orbit	Nivolumab	3 mg/kg every 2 wks, 13 cycles	Partial regression, Alive with stable disease	6.5	None
		69	M	Temple skin	Excision, irradiation, leuprolide injections	Local recurrence with extension to orbit	Ipilimumab	3 mg/kg every 2 wks, 4 cycles	Switch to pembrolizumab owing to progression	2	None
				Pembrolizumab	10 mg/kg every 3 wks, 28 cycles	Alive with stable disease	30	Dermatitis
	
	

### PD-1 inhibitors: Nivolumab and Pembrolizumab

PD-1 is an inhibitory immune checkpoint receptor expressed on T-cells that has a role in downregulating the immune system and promoting self-tolerance by suppressing T-cell activity. PD-1 can bind to two ligands expressed on cancer cells, PDL-1 and PDL-2, causing various inhibitory events within T-cells, including decreased production of cytokines and enzymes and inducing stagnation of the cell cycle.^[12]^ PD-1 inhibition by monoclonal antibodies allows T-cells to elicit a sustained immune response against cancer antigens.^[12]^ Cao et al revealed that PD-1/PDL-1 inhibitor therapy might be effective in conjunctival melanoma.^[24]^ They found that 19% of conjunctival melanomas expressed PDL-1 (cut-off 5%) and that this expression was correlated with the presence of distant metastases and worse melanoma-related survival.^[24]^ Although PDL-1 expression on conjunctival melanoma cells is thought to be somewhat less than in cutaneous melanoma (30–35%), it could be a therapeutic indicator or have prognostic value, as is seen in cutaneous melanoma.^[25]^

Ford et al first described successful responses to PD-1 inhibitors, pembrolizumab and nivolumab, in a patient with metastatic conjunctival melanoma and two patients with metastatic cutaneous melanoma and orbital involvement.^[19]^ An additional case series from the same group reported durable positive responses to PD-1 inhibitors in five patients with metastatic conjunctival melanoma.^[20]^ Of the five patients, four were treated with nivolumab and had a complete response with no evidence of disease at 1, 7, 9, and 36 months after completing treatment, respectively.^[20]^ The other patient treated with pembrolizumab had an initial stable condition but progressed after 11 months of therapy.^[20]^ PD-1 inhibitors were administered intravenously, with nivolumab at a dose of 3 mg/kg every two weeks for 3–11 months and pembrolizumab at a dose of 2 mg/kg, every three weeks for 11 months. Treatments were mainly discontinued when patients had side effects or disease progression.^[20]^ There have been several other case reports of pembrolizumab therapy for recurrent conjunctival melanomas as locally advanced or metastatic disease, which showed that pembrolizumab therapy can be used in patients who relapsed or metastasized after initial treatments such as surgical resection, cryotherapy, radiotherapy, exenteration, or ipilimumab.^[21,22,26,27]^

### CTLA-4 inhibitors: Ipilimumab

CTLA-4 is another negative regulator expressed on T-cells, interacting with ligands on antigen-presenting cells, including cancers. CTLA-4 inhibitors can result in undisturbed T-cell activation to attack their target.^[12]^ Ipilimumab, a CTLA-4 inhibitor, was the first checkpoint inhibitor approved for treating metastatic melanoma in 2011 after it showed improved overall survival compared with glycoprotein 100 peptide vaccine in patients with previously treated metastatic cutaneous melanoma.^[28]^ There have been a few case reports in the literature about ipilimumab used in combination with anti-PD1 checkpoint inhibitors for the treatment of metastatic or locally advanced conjunctival melanoma [Figure 2A & 2B].^[21]^

Sagiv et al reported a case of conjunctival melanoma with lung metastasis.^[20]^ The patient was initially treated with pembrolizumab but showed progression of metastatic disease after the 11-month treatment.^[20]^ Therapy was switched to intravenous ipilimumab at 3 mg/kg, combined with intravenous dacarbazine at 800–1000 mg/m^2^, every three weeks.^[20]^ The metastases showed partial improvement after two cycles of ipilimumab, but the treatment was stopped due to hepatotoxicity.^[20]^ Chaves et al described a case of locally advanced conjunctival melanoma with nodal metastasis.^[29]^ The patient was treated with adjuvant ipilimumab at 3 mg/kg every three weeks for four cycles, after surgical debulking and brachytherapy.^[29]^ No adverse effect of immunotherapy was reported in this single case report, and the patient had no evidence of local recurrence or metastasis at 16 months of follow-up.^[29]^

### Adverse events associated with immune checkpoint inhibitors

Immune checkpoint inhibitors enhance T-cell response against cancer cells but can also cause an autoimmune response against normal tissues. Adverse events associated with immune checkpoint inhibitors include immune-mediated pneumonitis, colitis, hepatitis, nephritis, skin problems such as pruritis and rash, and thyroid dysfunction.^[25,30]^ Large-scale studies in patients with cutaneous melanoma reported that grades 1–2 adverse events, such as diarrhea, pruritis, rash, and fatigue, occurred in about 19–36% of patients treated with immune checkpoint inhibitors.^[31,32]^ More severe adverse events of grades 3–5 were reported in 10% of pembrolizumab-treated cases, 22% of nivolumab-treated cases, 28% of ipilimumab-treated cases, and 59% of combined ipilimumab- and nivolumab-treated cases.^[25][31,32]^ If adverse events develop following immunotherapy, this usually leads to the discontinuation of the therapy, either temporarily or permanently, or to additional treatment with steroids or antihistamines.^[25]^

##  CONCLUSION

Conventional treatment of conjunctival melanoma has relied on surgical excision with adjuvant therapy, including cryotherapy, radiotherapy, and topical or systemic chemotherapy. There has been no consensus on the optimal treatment options for locally advanced or metastatic conjunctival melanoma. Historically, radical disfiguring and highly morbid surgeries, such as orbital exenteration, were done as the only treatment alternative for locally advanced recurrent conjunctival melanoma, yet no survival benefit is seen with orbital exenteration. Recent advances in immunotherapy have successfully allowed novel systemic agents to have a role in primary treatment, adjuvant therapy, or as an alternative to surgery in patients with locally advanced or multiply recurrent conjunctival melanoma; and they have also been proven effective in the treatment of metastatic conjunctival melanoma. The effectiveness and safety of immunotherapy for patients with locally advanced or metastatic conjunctival melanoma should be further elucidated in future studies. Ophthalmic surgeons should be aware of the potential role of immunotherapy in such patients

##  Financial Support and Sponsorship

None.

##  Conflicts of Interest

None.
